# Clinical profile and management of non-tubal ectopic pregnancy: Experience from a tertiary care hospital in the United Arab Emirates (UAE)

**DOI:** 10.12669/pjms.40.9.8584

**Published:** 2024-10

**Authors:** Shumaila Aftab Khan, Shaba Molvi, Giya Mathew, Muna Khalfan

**Affiliations:** 1 Dr. Shumaila Aftab Khan, FCPS, MRCOG. Department of Obstetrics & Gynaecology, Altnagelvin Hospital, Western Health & Social Care Trust, Londonderry, Northern Ireland, United Kingdom. Department of Obstetrics & Gynecology, Al Qassimi Women’s & Children’s Hospital, Emirates Health Services, Sharjah, United Aab Emirates; 2 Dr. Shaba Molvi, MD. Department of Obstetrics & Gynecology, Al Qassimi Women’s & Children’s Hospital, Emirates Health Services, Sharjah, United Aab Emirates; 3 Dr. Giya Mathew, MS, DNB, MRCOG. Department of Obstetrics & Gynecology, Al Qassimi Women’s & Children’s Hospital, Emirates Health Services, Sharjah, United Aab Emirates; 4 Dr. Muna Khalfan, Saudi & Arab Board. (Fetal Medicine Consultant, Head of Dept. OBGYN), Department of Obstetrics & Gynecology, Al Qassimi Women’s & Children’s Hospital, Emirates Health Services, Sharjah, United Aab Emirates

**Keywords:** Non-tubal ectopic pregnancy, Scar pregnancy, Cornual pregnancy, Cervical pregnancy, Ovarian pregnancy

## Abstract

**Objective:**

To study the clinical profile and management outcomes of non-tubal ectopic pregnancy at a tertiary care hospital in the United Arab Emirates (UAE).

**Methods:**

Case files of non-tubal ectopic pregnancy (NTEP) patients from October 2017 to October 2020 presented to Alqasmi Women and Children’s Hospital, Sharjah, were included in the study. The data was extracted from available medical records.

**Results:**

A total of 30 confirmed cases of NTEP were identified with the following break-up: caesarean scar ectopic pregnancy (n=14; 46.7%), cornual pregnancy (n=11; 36.7%), cervical pregnancy (n=3; 10%) and ovarian pregnancy n=2; 6.7%). Abdominal pain was the most common presenting symptom, present in 23 (76.7% patients) followed by vaginal bleeding in 17 (56.7%) patients. Ten women were managed medically, ten required surgical treatment, and eight were managed with combined medical and surgical treatments; Two patients were managed expectantly. Patients in each treatment strategy did well and there were no deaths. One patient in the medical treatment group developed complications after one month and underwent subtotal hysterectomy.

**Conclusion:**

Patients with NTEP were presented with varying symptoms and signs depending upon the location of NTEP. The different currently available management options for NTEP seem to be effective and safe when carefully selected based on the clinical merits of each case.

## INTRODUCTION

Non-tubal ectopic pregnancy (NTEP) is the implantation of an embryo at a site lying outside the uterine cavity or fallopian tubes. NTEP is a rare but potentially life-threatening condition. The incidence of ectopic pregnancy is estimated at about 1% to 2% of which about 5.0% to 8.3% are estimated to be NTEP. Risk factors for ectopic pregnancy include tubal damage following surgery or pelvic infection disease (PID), smoking and in vitro fertilization. However, the majority of women with an ectopic pregnancy have no identifiable risk factor.^1^,^2^

NTEP may implant in the cervix, uterine cornu, ovary, abdominal cavity or Caesarean section scar and pose significant diagnostic and management dilemmas. The most common symptom of NTEP is vaginal bleeding, which is often profuse and painless. The major complications of NTEP include the high risk of hemorrhage and potential loss of fertility. While serial beta human chorionic gonadotrophin (β-hCG) levels are commonly used to monitor early pregnancies, the ultrasound findings of a gestational sac are essential to make a diagnosis of NTEP.^3^

The management for NTEPs has wide variations.^4^ Traditionally NTEPs have been managed by open surgery. The described surgical treatment modalities include suction curettage, hysteroscopic resection or vessel coagulation, laproscopic/laparotomy wedge resection, balloon tamponade and hysterectomy.^3^,^5^ Surgical management is associated with increased morbidity and potential loss of fertility. Advances in diagnostics and increasing availability of first trimester ultrasound and quantitative β-hCG measurements have resulted in earlier diagnosis of ectopic pregnancies including NTEP leading to increasingly feasible to manage these cases medically, with associated reduction in morbidity and preservation of fertility.^6^,^7^ For most patients, medical management, is primarily through systemic and/or local methotrexate (MTX) administration. Some patients with NTEP may need a combination of surgical and medical management while still others may need only observation.

There is very little data available about the clinical profile management and outcome of NTEP in the United Arab Emirates. In this present manuscript, we report the clinical profile and management outcomes of NTEP at a tertiary care hospital in the UAE.

## METHODS

Consecutive patients of NTEPs who were retrospectively identified from the Early Pregnancy Assessment unit (EPAU) Alqasmi Women & Children’s Hospital database between October 2017 to October 2020. Patients with a suspected NTEP had their medical records reviewed and subsequently included in the study only if there was a confirmed NTEP diagnosis by either positive serum β -hCG and transvaginal ultrasonography findings consistent with NTEP and/or intra-operative and tissue histopathology diagnosis. Ultrasound diagnosis was made on established diagnostic criteria for each of the nontubal sites,^8^,^9^ shown in appendix- I. All patients monitored by serial BHCG levels every 48 hours showed decreasing trend later followed by weekly monitoring till it tested negative.

**Appendix I:** Ultrasound features of non-tubal ectopic pregnancy by site.^1^,^8^

Type of ectopic pregnancy	Ultrasound features
Cornual/Rudimentary horn pregnancy	i) The presence of a single interstitial portion of fallopian tube in the main uterine body of a unicornuate uterus, with (ii) the products of conception completely enveloped by myometrium, yet mobile and separate from the uterus, and (iii) a vascular pedicle adjoining the gestational sac to the unicornuate uterus
Caesarean Scar	(i) An empty uterine cavity and (ii) an empty endocervical canal, and (iii) a gestational sac implanted anteriorly at the level of the internal os, with (iv) a thin/absent layer of myometrium between the gestational sac and the bladder, and (v) evidence of significant trophoblastic/placental circulation
Cervical	i) An empty uterine cavity, with (ii) a barrel-shaped cervix and (iii) a gestational sac present below the level of the internal cervical os, with (iv) a negative ‘sliding sign’, and (v) blood flow around the gestational sac using colour Doppler
Ovarian	i) An empty uterine cavity with (ii) a wide echogenic ring with an internal anechoic area on the ovary that is (iii) with a negative ‘sliding sign’. A gestational sac with the fetal pole, yolk sac or even fetal heart activity may be seen within the ovary

Patients’ demographics, gestational age, symptoms at presentation, β-hCG level at presentation, ultrasound findings at diagnosis, clinical management provided, and complications were recorded for each case using a predefined proforma. NTEP were classified by the site of ectopic as well as by management provided. The sites were previous cesarean scar, cervix, cornua and ovary. The primary treatment modalities were classified as expectant, medical, surgical and combined medical & surgical management. Primary expectant management (EM) was considered in patients with early gestational age, low levels of β-hCG, absence of fetal cardiac activity and hemodynamic stability. Primary medical management included treatment with systemic methotrexate (MTX) consisted of multiple doses (four doses) of MTX 50 mg/m2 intramuscularly each. Selection criteria for medical management of ectopic pregnancy was described in appendix- II.^10^

**Appendix II:** Selection criteria for medical management of tubal pregnancy.^10^

Criteria	Consider MTX	Use MTX with caution	Use MTX with extreme caution
Vital signs	Normal	–	Abnormal
Abdominal pain	None	Mild/transient	Significant or persistent
b-hCG	<1500 IU/L	1500-5000 IU/L	>5000 IU/L
Adnexal mass size	<35mm	–	>35mm
Appearance	Empty gestational sac or heterogeneous mass	Yolk sac with or without fetal pole	Fetal heart rate seen
Free fluid on ultrasound	None/minimal	Simple and/or confined to pelvis	Echogenic or large
Ability to follow-up	No identified barriers agreeable to follow-up	Potential barriers	Unable or unwilling to follow up
Labs	Normal CBC, Cr, ALT	Mild anemia or thrombocytopenia Slight elevation in ALT or Cr (no more than 2 x upper limit of normal)	Significant abnormalities in any of CBC, Cr, ALT

Primary surgical management included (a)Laparotomy/laparoscopy and excision of ectopic pregnancy with repair of Cornue/ovary was considered in those women who were hemodynamically unstable or had evidence of intra-abdominal bleeding. Those who were hemodynamically stable with no evidence of intra-abdominal bleeding underwent (b) ultrasound guided (USG) suction evacuation / diagnostic hysteroscopy followed by suction evacuation.

Primary combined medical & surgical treatment was provided to patients based on their clinical assessment and ruling out of any contraindication to medical treatment. It consisted of a single dose of methotrexate 50 mg/m^2^ intramuscularly, ultrasound-guided intra-sac injections (USGI) of feticide potassium chloride followed by USG suction evacuation with or without prophylactic bilateral internal iliac artery balloon occlusion.

Patients remained hospitalized until the effect of treatment was proven by decreasing trend of serial β-hCG level thereafter followed by weekly monitoring till it became undetectable.

### Statistical Analysis:

Data was de-identified and analyzed using IBM SPSS Statistics 23. The categorical variables are presented as frequency (percentage), while continuous variables are presented as mean±standard deviation (SD).

### Ethical approval:

The study was approved by the ministry of health and prevention research ethics committee. This was a retrospective study; the waiver of the informed consent was obtained. (MOHAP/DXB-REC/ OON/No.149 /2020).

## RESULTS

In this manuscript, 30 confirmed NTEP cases were identified & were classified by the sites and management provided. CSEP was the most common type of NTEP, accounting for nearly half of all cases.

The mean age of the patients at diagnosis was 32.6±4.95 years (range 23-42 yrs.). The mean Hb level at admission was 11.08±1.6 g/dl the β-hCG level at admission was 23,704.4 ± 30295.8 (mIU/ml) while the β-hCG level on discharge was 46.500± 155.903 (mIU/ml). The average gestational age at diagnosis was six weeks three days (range six weeks to 13 weeks).

Previous cesarean section was the commonest risk factor, present in 70% of the patients. All NTEP were conceived spontaneously except three patients (10%) had fertility enhancing treatment. Only one patient had a history of PID. Abdominal pain was the most common presenting symptom (77 %), while vaginal bleeding and maternal shock were found in 57% and 13.3% of patients, respectively. The 26 patients (86.3 %) were hemodynamically stable on presentation. In four cases no symptoms were recorded. The clinical characteristics of cases in different management groups are shown in Table-I.

**Table-I:** Patient characteristics in different groups of NTEP patients (n=30).

	Cornual	Scar	Cervical	Ovarian
Number, n (%)	11 (30.0)	14 (30.0)	3 (6.7)	2 (33.3)
Age, years	31.3 ± 5.02	33.9 ± 5.36	33.3 ± 3.51	29.5 ± 0.71
Hb level on admission, g/dl	10.5 ± 2.20	11.5 ± 1.15	11.7 ± 0.44	10.6 ± 2.05
β-hCG level on admission, mean (IU/L)	19816.9±18738.7	27833.4±39953.9	31077.3±23290.3	5124.0±3227.2
β-hCG level on discharge, mean (IU/L)	2.27 ± 6.07	95.6 ± 221.9	10.3 ± 14.6	0.00 ± 0.00
Gestational age at diagnosis (days)	45 ± 18.7	54 ± 16.3	49 ± 0.57	24 ± 34.6
***Risk factors:***				
Prior cesarean section n=21 (70 %)	4 (36.4%)	14 (100%)	2 (66.7%)	1 (50%)
Use of fertility enhancing treatment n= 3 (10%)	2 (66.7%)	0 (0.0%)	1 (33.3%)	0 (0.0%)
Advanced maternal age n=10 (33.3 %)	3 (30.0%)	6 (60.0%)	1 (10.0%)	0 (0.0%)
PID n =1 (3.33 %)	1 (100%)	0 (0%)	0 (0%)	0 (0%)
***Symptoms:***				
Abdominal pain, n =23 (76.7 %)	7 (30.4%)	12 (52.2%)	2 (8.69%)	2 (8.69%)
Vaginal bleeding, n=17 (56.6%)	8 (47.1%)	9 (52.9%)	0 (0%)	0 (0%)
Dizziness, n=9 (30%)	3 (33.3%)	6 (66.7%)	0 (0%)	0 (0%)
Shoulder tip pain, n =2 (6.66 %)	1 (50.0%)	1 (50.0%)	0 (0%)	0 (0%)
Breathlessness, n =2 (6.66%)	0 (0%)	2 (100.0%)	0 (0%)	0 (0%)
Maternal Collapse, n=4(13.3%)	3 (75%)	1 (25%)	0 (0%)	0 (0%)

The continuous variables are represented as Mean ±SD while the categorical variables are represented as frequency (percent). “Combined” means both medical and surgical management. Hb, hemoglobin; β-hCG, beta human chorionic gonadotrophin; SD, standard deviation. NTEP, non-tubal ectopic pregnancy; PID, pelvic inflammatory disease.

The line of treatment selected by the doctors was derived from clinical judgment. Based on the site of implantations, patients with cornual pregnancy (Cop) were treated almost equally by surgical or medical treatment. On the other hand, most of the patients with scars (CSEP) or cervical sites pregnancy (CP) were managed with combined medical & surgical treatment. Ovarian pregnancies (OvP) were managed by primary surgical treatment (laparotomy/laparoscopy) as all presented with acute abdomen and were hemodynamically unstable.

In this study, two patients were managed by expectant management, ten women received medical management, ten had surgical management and eight women were managed by combined treatment (Table-II). The main outcome measured was the success of each primary treatment modality Table-III. Primary management was considered successful if no further changes in treatment modality were required and if serial β-hCG levels dropped significantly.

**Table-II:** Primary management strategies according to the site of implantation of NTEP.

Primary management strategy	Site of implantation			

	Cornual pregnancy	Scar pregnancy	Cervical pregnancy	Ovarian pregnancy	Total
Expectant	1	1	0	0	2
Medical	4	4	2	0	10
Surgical	6	2	0	2	10
Combined	0	7	1	0	8
Total	11	14	3	2	30

NTEP, non-tubal ectopic pregnancy.

**Table III:** Outcome in NTEP as per the type of management.

Management Outcomes	Type of primary Management				

	Medical=10	Surgical=10	Combined=8	Expectant =2	Total
Hemorrhage (1000 ml)	1	1	0	0	2
Blood transfusion	1	6	0	0	7
Infection	1	0	0	0	1
Cesarean Section Scar rupture	1	0	0	0	1
Injuries to cervix	0	0	0	0	0
ICU admission	1	3	1	0	5
Prolonged hospitalization*	1	3	4	0	8
Hysterectomy	1	0	0	0	1
Satisfactory outcome	9 (90%)	10 (100%)	8 (100%)	2 (100%)	29 (96.7%)

ICU, intensive care unit; NTEP, non-tubal ectopic pregnancy.

* Hospitalization for >3 days.

EM approach was adopted in two cases of CSEP and CoP, in consideration of clinical stability, early gestational age, low level of β-hCG and absence of fetal cardiac activity. During the recovery, the patient had a complete miscarriage. After three days, all the patients were discharged in good physical condition; the follow-up with ultrasound scan and biochemical testing after 1-4 weeks, did not detect any complications.

Primary medical management was successful in seven patients proved by significant β-hCG drop with no complications, but one case was reported unsuccessful. This was a CSEP that received primary medical management based on standard criteria and was discharged in good clinical condition with a decrease in serum β-hCG from value of 97.00 IU to 33.00 IU within 48 hours with further follow up advice. The patient was readmitted after five days as she developed fever, vaginal bleeding and suspected infection with a low β-hCG value of 30 IU. She underwent USG suction evacuation and discharged after a couple of days of being asymptomatic. A month after the procedure, she was once again presented in A&E with profuse vaginal bleeding with very low β-hCG level of 2.0 IU. She ultimately ended up with an emergency subtotal hysterectomy and bilateral salpingectomy as lifesaving procedure.

The primary surgical management group included six CoP, two OvP, and two CSEP. In this group, those patients who were presented with acute abdomen with evidence of intra-abdominal bleeding underwent emergency laparotomy/laparoscopy and excision of ectopic pregnancy with repair of cornue/ovarian reconstruction. Both OvP, hemodynamically unstable at time of presentation, were also treated by laparotomy with ovarian wedge resection/reconstruction. While two CSEP in this group were hemodynamically stable and had USG suction evacuation. No postoperative complications were observed at the one week and four weeks of follow-up. Admission to the intensive care unit was necessitated in three patients in the surgical group, these were the patients who had hemoperitoneum at presentation and received blood transfusions.

**Fig.1:**
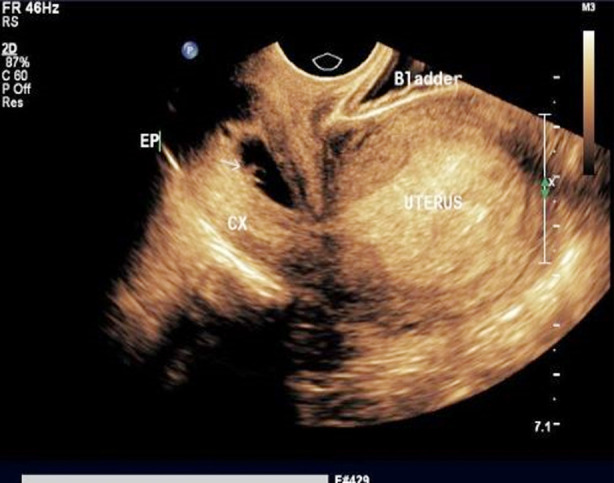
Transvaginal image (2D) of cervical pregnancy. A gestational sac (GS) with a fetal pole is seen inside the cervix.

In the combined medical & surgical treatment group, seven patients with CSEP and one with CP (Fig.1) were treated successfully with no reported complications. Prophylactic bilateral internal iliac artery balloon occlusion was performed in three patients. Overall, the length of hospital stay was reported more in patients who were treated surgically or with combined treatment.

## DISCUSSION

NTEPs represent an important challenge for the gynecologist because of the rarity of the disease and the lack of consensus regarding treatment strategies or protocol.^4^ The widespread availability and application of ultrasound have helped gynecologists in the early identification and localization of the gestational sac and in choosing the appropriate management of ectopic pregnancies. The availability of dedicated early pregnancy assessment units (EPAUs) has further improved diagnosis and follow-up for these patients.^6^ In the present study, we report data from a three-years retrospective review of all diagnosed cases of NTEPs and their subsequent management at a tertiary care hospital in the Middle East.

In the largest reported series, the common epidemiological factors associated with the development of NTEP were pelvic inflammatory disease and assisted reproductive techniques.^5^ In this series, scars from a previous cesarean section and advanced maternal age were the most common risk factors identified while only one patient had a history of PID. Three patients had fertility-enhancing therapies. No specific risk factor was identified in a significant proportion of the patients.

In this study, the most common site of implantation of NTEP was a previous cesarean scar and uterine cornu. The clinical presentation of NTEP is quite variable. While in some patients the diagnosis is made incidentally, others may be quite sick from acute internal hemorrhage. In one of the previous studies, the most common symptoms of NTEP were abdominal pain, vaginal bleeding, or both.^5^ In this study, the most common clinical presentation in different groups was abdominal pain 76% (23 patients). The next common clinical presentation was vaginal bleeding 56.6% (17 patients). In general, ovarian NTEP was most likely to present with intraperitoneal bleeding while CSEP had a mild symptom. In this study, the findings agree with previously published reports about NTEP.

Owing to the rarity of NTEP, there is no consensus regarding treatment strategies or protocol. The type of treatment strategy depends on the site, local expertise, resources and patient characteristics.^10^,^11^ Expectant management does not seem to be an appropriate choice for most cases and maybe only suitable option for clinically stable women with declining β-hCG levels and absence of fetal cardiac activity.^9^ In this study, two patients were managed successfully by expectant management (CoP and CSEP).

In terms of available management options for NTEP, surgical management (laparotomy/laparoscopy) is indicated in women with hemodynamic compromise or other clinical signs of ruptured NTEP including abdominal pain or evidence of intra-abdominal bleeding, contraindications to medical treatment, and according to patient preference.^9^,^12^ In this study four (13.3%) patients presented with acute abdomen and were hemodynamically unstable; were managed by emergency laparotomy. Those who were hemodynamically stable but showed any evidence of intra-abdominal bleeding also underwent laparotomy/laparoscopy. USS/hysteroscopy guided suction evacuation was performed in patients who were hemodynamically stable with no evidence of intra-abdominal bleeding.

Primary medical management with Sys-MTX is also one of the well-recognized treatment options in hemodynamically stable patients. SOGC Guideline recommends methotrexate is a safe and effective treatment for carefully selected tubal and NTEP.^10^ In this study, ten patients were managed with medical management successfully.

Tuncay and colleagues successfully treated NTEP by local MTX in one case series.^13^ One local study reported an ultrasound guided transvaginal aspiration and injection of 50 mg MTX followed by needle mechanical disruption as an effective treatment approach for CSEP.^14^

Several authors in recent years have described MTX appears more efficient when combined with curettage or hysteroscopy. One small case series had reported a successful laparoscopic guided transcervical evacuation and hysteroscopic approach in unruptured interstitial pregnancies.^15^

This study indicates that a treatment combining MTX, and suction evacuation appears to be effective and safe in pregnancies with early gestational age. In this study seven CSEP and one CP were managed with a combined medical and surgical approach successfully.

## CONCLUSION

We conclude that patients with NTEP were presented with varying symptoms and signs depending upon the location of NTEP. In general, the most common clinical presentation in NTEP in this study was abdominal pain followed by vaginal bleeding. Ovarian NTEP was most likely to present with intraperitoneal bleeding while CSEP had a milder course. Most of the patients were managed based on individual clinical assessment and the prognosis was generally good.

The present study supports the idea that the treatment should include all the available options: expectant, medical, surgical, combination (medical & surgical) management after individual assessment of each NTEPs.

### Authors’ Contribution:

**SAK** conceived, designed, manuscript writing and responsible for integrity of research.

**GM, SM** did data collection, analysis and review of the manuscript.

**MK** helped in design, Analysis and review of the manuscript.

## References

[1] Diagnosis and Management of Ectopic Pregnancy:Green-top Guideline No. 21 (2016). BJOG.

[2] Long Y, Zhu H, Hu Y, Shen L, Fu J, Huang W (2020). Interventions for non-tubal ectopic pregnancy. Cochrane Database Syst Rev.

[3] Ash A, Smith A, Maxwell D (2007). Caesarean scar pregnancy. BJOG.

[4] Shah JS, Nasab S, Papanna R, Chen HY, Promecene P, Berens P (2019). Management and reproductive counseling in cervical, caesarean scar and interstitial ectopic pregnancies over 11 years:identifying the need for a modern management algorithm. Hum Reprod Open.

[5] Ng S, Hamontri S, Chua I, Chern B, Siow A (2009). Laparoscopic management of 53 cases of cornual ectopic pregnancy. Fertil Steril.

[6] Loh WN, Adno AM, Reid S (2022). A 10-year retrospective cohort study of non-tubal ectopic pregnancy management outcomes in an Australian tertiary centre. Australas J Ultrasound Med.

[7] Delplanque S, Le Lous M, Flévin M, Bauville E, Moquet PY, Dion L, Fauconnier A, Guérin S, Leveque J, Lavoué V, Nyangoh Timoh K (2020). Effectiveness of conservative medical treatment for non-tubal ectopic pregnancies:a multicenter study. J Gynecol Obstet Hum Reprod.

[8] Kirk E, Ankum P, Jakab A, Le Clef N, Ludwin A, ESHRE working group on Ectopic Pregnancy (2020). Terminology for describing normally sited and ectopic pregnancies on ultrasound:ESHRE recommendations for good practice. Hum Reprod Open.

[9] Jabeen K, Karuppaswamy J (2018). Non-surgical management of caesarean scar ectopic pregnancy- a five-year experience. J Obstet Gynaecol.

[10] Po L, Thomas J, Mills K, Zakhari A, Tulandi T, Shuman M (2021). Guideline No. 414:Management of Pregnancy of Unknown Location and Tubal and Nontubal Ectopic Pregnancies. J Obstet Gynaecol Can.

[11] Timor-Tritsch IE, Monteagudo A (2012). Unforeseen consequences of the increasing rate of cesarean deliveries:early placenta accreta and cesarean scar pregnancy. A review. Am J Obstet Gynecol.

[12] Tremmel E, Starrach T, Buschmann C, Trillsch F, Kolben T, Mahner S (2024). Management of non-tubal ectopic pregnancies analysis of a large tertiary center case series. Arch Gynecol Obstet.

[13] Tuncay G, Karaer A, Coskun EI, Melekoglu R (2018). Treatment of unruptured cornual pregnancies by local injections of methotrexate or potassium chloride under transvaginal ultrasonographic guidance. Pak J Med Sci.

[14] Bassam MA, Begam MA, Aswad SG, Al-Jefout M (2019). Ultrasound guided transvaginal aspiration and mechanical destruction with local methotrexate injection is a promising primary treatment approach for caesarean scar ectopic pregnancy (CSEP). Biomed Pharmacol J.

[15] Kahramanoglu I, Mammadov Z, Turan H, Urer A, Tuten A (2017). Management options for interstitial ectopic pregnancies:A case series. Pak J Med Sci.

